# Supplementary material for: Acceptability determinants of a proposal to reduce antiretroviral treatment to an oral two-drug regimen among patients living with HIV and physicians in France
Source: PLoS One. 2024 Sep 30;19(9):e0308784. doi: 10.1371/journal.pone.0308784 (PMC11441690; doi:10.1371/journal.pone.0308784)
Supplement: S1 File — (DOCX) [file pone.0308784.s001.docx]

| Anonymisation code | Gender | Location of practice | Age | Year of thesis | Years of HIV practice | Number  of HIV  patients | Speciality | Hospital /University position |
| --- | --- | --- | --- | --- | --- | --- | --- | --- |
| 001-001-RP | M | Ile-de France | 34 | 2015 | 4 | 300 | Infectious diseases | HP^[[1]](#footnote-1)^ |
| 002-001-MS | M | Ile-de France | 37 | 2014 | 12 | 250 | Infectious diseases | HP |
| 003-001-TC | M | Ile-de France | 36 | 2018 | 3 | 85 | General medicine | HP |
| 004-001-DR | M | Province | 63 | 1987 | 30 | 350 | Internal medicine | HP |
| 005-001-CM | F | Ile-de-France | 56 | 1994 | 26 | 140 | General medicine | HP |
| 006-001-JL | M | Ile-de-France | 47 | 2003 | 17 | 200 | Infectious diseases | HP |
| 007-001-VG | F | Ile-de-France | 55 | 1995 | 25 | 500 | Infectious diseases | HP |
| 008-001-SA | F | Province | 55 | 1998 | 20 | 120 | Infectious diseases | UP-HP^[[2]](#footnote-2)^ |
| 011-001-PS | M | Ile-de-France | 55 | 1994 | 26 | 1000 | Infectious diseases | HP |
| 012-001-PP | M | Province | 67 | 1980 | 40 | 100 | Internal medicine | HP |
| 013-002-DM | F | Province | 58 | 1993 | 29 | 40 | Internal medicine | HP |
| 014-001-IL | F | Province | 58 | 1991 | 36 | 300 | Internal medicine | HP |
| 015-001-HL | F | Province | 34 | 2015 | 5 | 200 | General medicine | HP |
| 015-002-OF | F | Province | NI | NI | NI | NI | Infectious diseases | HP |
| 016-001-NH | F | Province | 36 | 2013 | 8 | 100 | Internal medicine | HP |

S1 Table. Description of physicians

| Anonymisation code | Gender | Location of follow-up | Year of diagnosis | Current treatment | Attitude to change |
| --- | --- | --- | --- | --- | --- |
| E1-PB | M | Province | 2005 | Oral bitherapy | Adhere^[[3]](#footnote-3)^ |
| E2-CP | M | Ile-de France | 2007 | Tritherapy – 4/7 | Adhere |
| E3-JB | F | Ile-de France | 1991 | Tritherapy – 7/7 | Hesitant^[[4]](#footnote-4)^ |
| E4-CP | M | Ile-de-France | 2019 | Tritherapy – 7/7 | Opposed^[[5]](#footnote-5)^ |
| E5-FM | F | Ile-de-France | 1989 | Tritherapy – 7/7 | Adhere |
| E6-JT | M | Ile-de-France | 1987 | Tritherapy – 7/7 | Hesitant |
| E7-JD | F | Ile-de-France | NI | Tritherapy – 7/7 | Adhere |
| E8-JB | F | Ile-de-France | 1993 | Tritherapy – 7/7 | Hesitant |
| E9-SK | M | Ile-de-France | 2007 | Tritherapy – 7/7 | Hesitant |
| E10-VB | F | Ile-de-France | 1996 | Tritherapy – 4/7 | Opposed |
| E11-PP | F | Province | 1991 | Tritherapy – 7/7 | Opposed |
| E12-BA | M | Ile-de-France | 1996 | Oral bitherapy | Adhere |
| E13-YM | M | Province | 2012 | Oral bitherapy | Adhere |
| E14-RM | M | Province | 2018 | Oral bitherapy | Adhere |
| E15-SL | F | Ile-de-France | 2011 | Oral bitherapy | Hesitant |

S2 Table. Description of patients


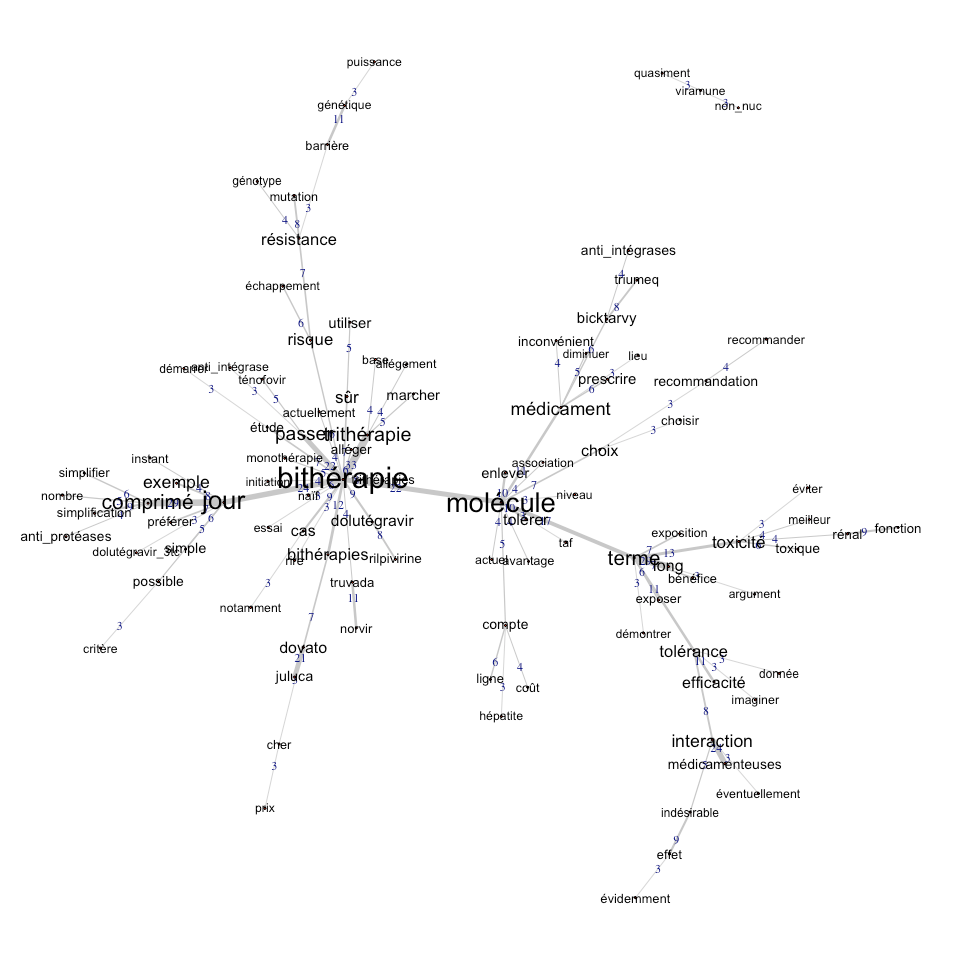


S3 Fig. Similarity analysis on the word bitherapy


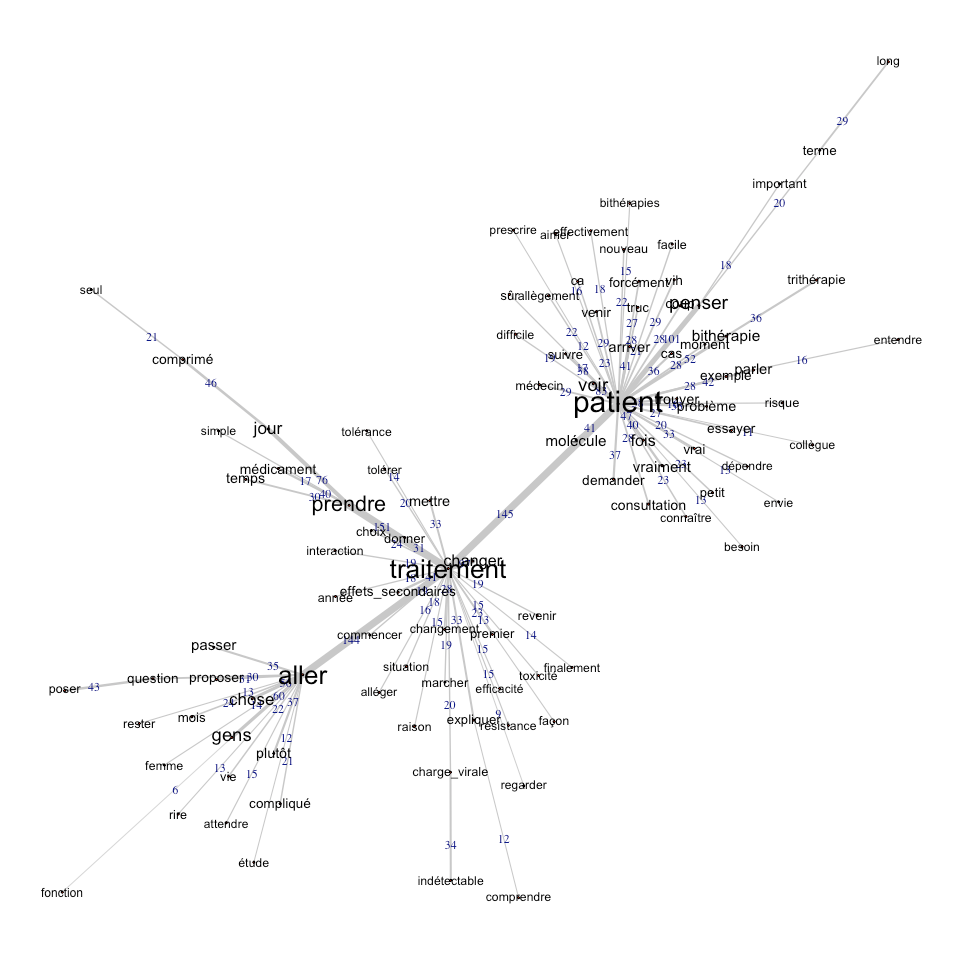


S4 Fig. Similarity analysis of total corpus


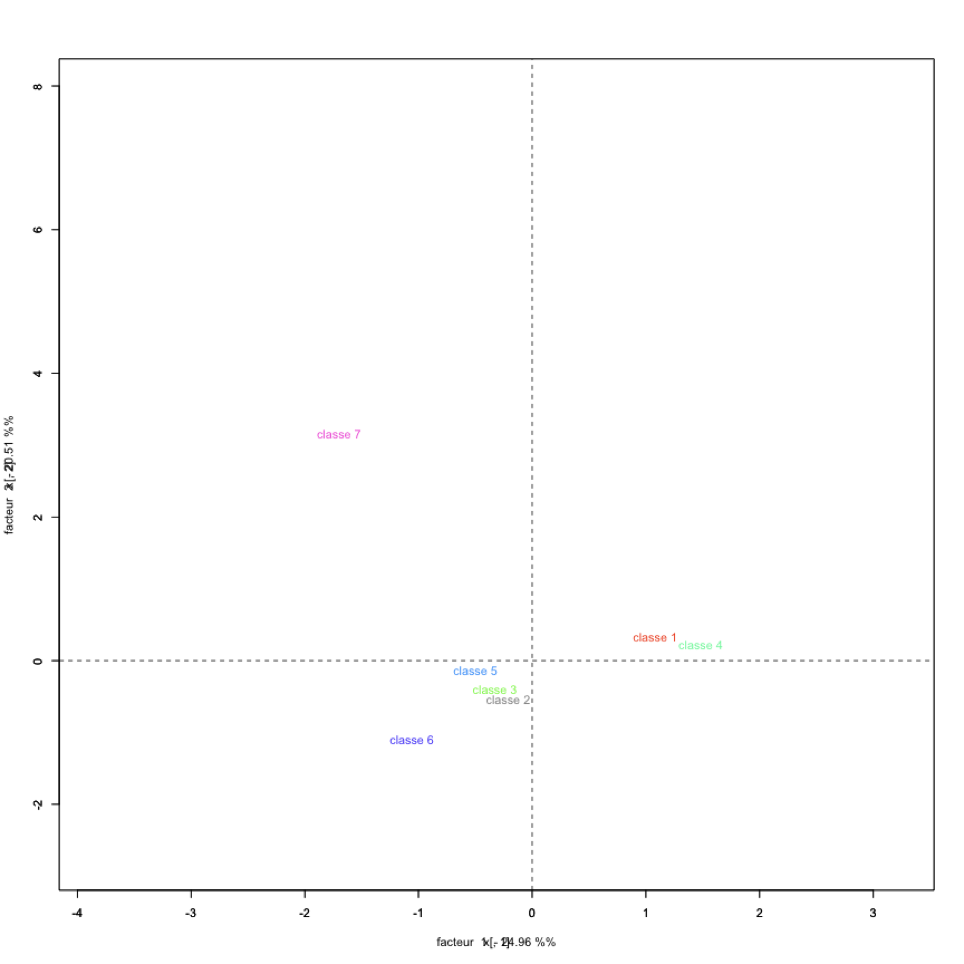


S5 Fig. Correspondence factor analysis

| **Themes discussed with physicians** | **Quotations** | **Themes discussed with PLWH** | **Quotations** |
| --- | --- | --- | --- |
| (1) Relationships with patients | «And then these are patients who again, in their history, for whom it may have been difficult to get an undetectable viral load, to find a treatment that works. There is the resistance to change that is common to everyone, the fear, the experience, you live something again» (E4-C-M) | (1) HIV treatment initiation | «The side effects, because when you have an active life before, sport, work and everything stops, it's true that it's not easy. And then it's mostly that. It's 200% different, life is not the same at all” (E2-P-M) |
| (2) Treatment choice and practices at initiation | «So I choose the treatment according to the recommendations, the possible drug interactions, the possible comorbidities related to other health problems, for example renal insufficiency, hepatic insufficiency.» (E14-C-F) | (2) Experiences of treatment change | «I had several changes in treatment for mainly gastric reasons. It was always the side effects that caused me problems.» (E12-P-M) |
| (3) Treatment choice and practices during a change of treatment | «It's "a la carte", and it's really something I'm attached to, really» (E12-C-F) | (3) Perceptions of treatment mitigation strategies | «It reassures me because the purpose of this disease, at the beginning there were no drugs and today we have treatments and we live with » (E9-P-M) |
| (4) Treatment reduction practices | «There is a bit of an embarrassment of riches to choose from, so everyone has their own little habits, quotation marks. It is not at all obvious to prioritize or to say precisely that Bicktarvy I will give him to this profile, Delstrigo to that one, Dovato to such and such.» (E5-C-F) | (4) Perceptions of O2DR | «Dual therapy is more than two molecules instead of three, we are on two active molecules. That's always less for the liver, and it calms the kidneys down a bit. As long as it remains effective, there are no worries (E15-P-F) |
| (5) Perceptions of treatment reduction | We have quite a few ways of reducing the dosage, at least two of which are dual therapy or not taking the drug every day. So it's really a discussion, there's really a consideration of what the patient wants.» (E2-C-M) | (5) Experiences with tapering and oral combination therapy | «Well, I was a little apprehensive for the first two months to see the blood results, to see if it was working well. And it works well» (E1-P-M) |
| (6) Perceptions of O2DR | «So the advantage is less toxicity, the same efficiency, maybe less drug interactions» (E6-C-M) | (6) Non-HIV medication practices | «So I am not against allopathy in general and I am not against homeopathy or naturopathy, on the contrary » (E13-P-M) |
| (7) Proposals for switching to O2DR | «I take the image of shampoo, 3 in 1 or 2 in 1, so two molecules in one tablet is better than three for the same result.» (E15-C-F) | (7) Relationships with health professionals | «I am in complete confidence so he can offer me anything he wants, I am» (E3-P-F) |

S6 Table. Quotations of thematic analysis

| Name of the class | Interpretation | Quotations |
| --- | --- | --- |
| Class 1 : Treatment and monitoring | This class represents the determinants of overall HIV management, which includes factors such as adherence to treatment, the impact of HIV on daily life, and the importance of regular medical check-ups | « The consultations are always long but extremely complete, she follows a very rigorous methodology» (E12-P-M) |
| Class 2 : Social environment and support | This class focuses on the social support available to individuals managing HIV, including support from friends, family, and healthcare professionals. | « And so my family has always supported me. And I still have friends, not many, but always there to support me, and I have my ex-husband » (E5-P-F) |
| Class 3 : Lived experience and difficulties | This class highlights the individual difficulties, encountered both from the perspective of patients, in their experience of infection. But also among clinicians, the difficulties encountered in care. | « Because I was still in Africa and you know in Africa at that time, you never know if you are sick or not» (E7-P-M) |
| Class 4 : Sources of information about treatments | The class highlights the different sources of information mobilized by doctors in particular, about treatments. This class is directly related to the specific information questions proposed during the interview. | « Alors je dirais quand même que ma première source d’informations ce sont les congrès ou les réunions. Mais bon c’est sûr qu’en ce moment... il y a les visios. Mais donc les congrès, même si je n’y vais pas toutes les cinq minutes, j’essaye d’y aller assez régulièrement » (E12-C-F) |
| Class 5 : Communication about switch | This class represents the process of proposing a treatment change, with a focus on the interaction between physicians and patients. The presence of action verbs and the term "agreement" suggest that patient involvement in the decision-making process is important. | “ So I would say that my first source of information is congresses or meetings. But of course, at the moment... there are visio conferences. But even if I don't go often to the congresses, I try to go quite regularly» (E5-C-F) |
| Class 6 : Scientific vision of the switch | This class also represents medical discourse, specifically the medical vision of dual therapy. This highlights the importance of medical perspectives in the implementation of new treatment options. This class is characterized by medical discourse, specifically the criteria motivating the change of treatment. This suggests that medical factors play a significant role in treatment change decisions. | « The main thing for me for the patient is less toxicity in the long term. One molecule less, even if the current molecules are less toxic, there is less toxicity in the long term and this is the main thing for me» (E6-C-M) |
| Class 7 : Characteristics of HIV chronicity | This class focuses on the characteristics of chronicity, specifically the long-term impact of HIV on individuals' lives and the importance of adapting treatment to these changing circumstances. | « It’s like taking a Doliprane in the evening » (E3-P-F) |

S7 Table. Quotations of lexicometric analysis

1. HP : Hospital Physician [↑](#footnote-ref-1)
2. UP-HP : University Profesor – Hospital Physician [↑](#footnote-ref-2)
3. Adhere signifies that the participant's discourse is fully in favor of the dual therapy regimen. [↑](#footnote-ref-3)
4. Hesitant signifies that the participant is questioning or expressing uncertainty about the dual oral therapy regimen. [↑](#footnote-ref-4)
5. Opposed signifies that their statements clearly reflect a stance of opposition to the use of dual therapy. [↑](#footnote-ref-5)
