# Supplementary material for: Acceptability determinants of a proposal to reduce antiretroviral treatment to an oral two-drug regimen among patients living with HIV and physicians in France
Source: PLoS One. 2024 Sep 30;19(9):e0308784. doi: 10.1371/journal.pone.0308784 (PMC11441690; doi:10.1371/journal.pone.0308784)
Supplement: Attachment — Submitted filename: Response to reviewers.docx [file pone.0308784.s002.docx]

Review Comments to the Author

Please use the space provided to explain your answers to the questions above. You may also include additional comments for the author, including concerns about dual publication, research ethics, or publication ethics. (Please upload your review as an attachment if it exceeds 20,000 characters)

**Reviewer #1: line 52-54: Please elaborate the connection of this study to the PROBI qualitative study.**

The qualitative PROBI study takes place upstream of the quantitative PROBI study. It meets two objectives: the first one was investigated knowledge, beliefs and representations of O2DR among patients and physicians ; the second one was to create specific items within the PROQOL-HIV scale to measure the quality of life of patients who switched to O2DR.

**line 61-63: I think this should be described more straightforward to avoid confusion. In addition, the location of practice should be described in a simpler fashion, perhaps urban vs. rural, capital vs. provincial, etc, so that it's easier for readers not-so-familiar with France.**

Thank you for your feedback. We have simplified the description of the participants and added the location of the participants. The paragraph has been modified as follows: The study included interviews with adult patients living with HIV (PLWH) and physicians in France. PLWH were included if they were adults, fluent in French, and receiving antiretroviral (ARV) treatment. Patients were selected based on time since HIV diagnosis (less than 5 years vs. 5 years or more), gender, and type of ARV therapy (dual vs. triple or quadruple therapy). Physicians were eligible if they followed PLWH at least once a week and prescribed ARV treatment. They were selected to ensure diversity in terms of professional experience (young vs. older physicians), attitude towards dual therapy (favorable vs. reticent), and gender. Participants were recruited through a snowball effect until data saturation was reached. The interviews were anonymized using a unique code consisting of the participant's initials and a number. The study was conducted in both urban and rural settings across France

**Table 2: please consider presenting the summary instead of individual data and see which works best. Also, please provide explanation on abbreviations.**

We removed the tables presenting individual characteristics and presented the characteristics in summary form as follows. For professionals, the original Table 2, was summarized as follows: The data includes their gender, location of practice (either in the Île-de-France region or in a province), age, year of thesis, years of HIV practice, number of HIV patients they have treated, speciality, and hospital/university position.

For patients, the initial Table 3 was summarized as follows: The data includes their anonymization code, gender, location of follow-up (either in the Île-de-France region or in a province), year of diagnosis, current treatment, and their attitude towards changing their treatment.

Abbreviations have been explained. The two tables of individual participant characteristics have been placed in the appendix documents of the article.

**Table 3: would it be possible to include the patients' sociodemographic characteristics as well, e.g. education, employment status, etc. Also, consider presenting them in summary, not individual data.**

All sociodemographic data that we collected are now presented in the tables. We do not have data on education and employment. We deleted the individual data and replaced the tables with a summary of participant characteristics.

**Figure 1: this can be improved by adding, for instance: describe what class 1-7 refers to, put frequency (%) to the words, etc.**

The results of the lexicometric analysis were further described in the article. An appendix has been added to present each class with its name, the corresponding citations and the frequencies and occurrences of the words.

**Reviewer #2: First I would like to congratulate the authors on this manuscript. Thank you for the opportunity to review this work. This manuscript presents the results of a qualitative study investigating the determinants of the acceptance of a proposal to reduce antiretroviral treatment in patients living with HIV and physicians. This work brings to light very interesting results which could help better care for patients and relies on a double analysis: statistical text analysis and thematic analysis. However, the introduction is quite short and the statistical analysis appears quite secondary and should be better described/elaborated on. Similarly, I think the methods and interpretation part of this study should be better described to be fully understood and assessable.**

To provide a more comprehensive overview of our study, we agree with the reviewer that the introduction could benefit from more elaboration. Therefore, we have expanded the introduction to better describe the lexical analysis and methods used in the study. Additionally, we have provided more detail on the interpretation of the study's findings.

Major comments

Introduction
**1. "to date there lacks studies aimed at questioning the psychosocial determinants at stake in the context of treatment change" : Do the authors mean there is not any study on this topic? If so it should be worded that way.**

This was clarified. We meant to convey that while there have been some studies on the efficacy and safety of O2DR in terms of resistance and viral load maintenance, there is a lack of studies that specifically focus on the psychosocial determinants involved in treatment change. The sentence was revised to read: " to our knowledge, there are no studies that have specifically investigated the psychosocial determinants at stake in the context of treatment change."

**2. The introduction is not enough supported. In my opinion, the authors should develop it a bit more. I may suggest to discuss the literature on treatment adherence and/or shared decision making and/or other connected topics for example?**

Thank you for your feedback. We incorporated a discussion of the literature on treatment adherence and shared decision making to provide more context and support for our study.

Methods
**1. The authors specify they proceeded to a theoretical sampling logic. Were there any theoretical recruitment process (i.e., ideal proportion of participants according to each identified characteritic)?**

We employed a theoretical sampling logic, which is a non-probability sampling method based on selecting participants based on specific characteristics related to the research question. However, there is no mention of an ideal proportion of participants according to each identified characteristic. Although we did not expect to reach ideal proportions for each category but at least that all categories would be represented in the sample. The goal was to ensure a diverse range of perspectives on the phenomenon being studied. Participants were recruited through a snowball effect until data saturation was achieved.

**2. Similarly, their sampling was based on criteria thought of as indicators of the phenomena. How did the authors decided that these variables were indicators? This should be broached upon in the introduction.**

The criteria for participant selection in this study were selected based on a theoretical sampling logic, which incorporates criteria thought to be indicators of the phenomenon being studied. However, we agree that it would be helpful to provide more information on how these specific indicators were chosen. Therefore, we have added a sentence to the introduction that highlights the use of theoretical sampling and criteria selection in the study design : “For both populations, the sampling was based on case identification [14] followed by a theoretical sampling logic, incorporating criteria sought as indicators of the phenomenon in question”

**3. How was the professional experience dichotomized? What was the threshold? Why?**

Some professionals self-identified as either young or experienced clinicians during the interviews, and based on this information and the sample size, we made an arbitrary decision to dichotomize professional experience into those with less than 10 years of practice as young clinicians and those with more than 10 years of practice as experienced clinicians. This decision was based on the characteristics of our sample, and it allowed us to examine potential differences in perceptions and experiences between these two groups. However, it's important to note that this dichotomization was arbitrary, and other studies might use different thresholds or categories depending on the context of the research.

**4. The authors mention recruitment continued till data saturation. How was it assessed?**

We followed the principle of data saturation, which consists of stopping the data collection when no new themes or ideas emerge during the interviews. In the case of this study, the first ten interviews brought out new themes. From the eleventh interview until the 15th, the participants' discourse did not address any new themes.

**5. The interview guides were quite on the "directive side" of semi-structured interviewing, why did the authors choose to analyze conjointly the patients' and physicians' interviews? I am quite surprised for this choice as interview guide may both influence the topics told by participants and the word they use to tell their experience. Separate analyses may be a more sound choice.**

The analyses were not carried out jointly; each data collection was analyzed separately. The results presented in the article are the result of the methodological triangulation, where elements that cut across the discourses could be identified. This was specified in the method.

`
**6. P.3 The authors specify the variables taken into account in the Iramuteq analysis. What were the modalities of the "sexual orientation" variable for example? The results section do not present results pertaining to these variables. I advise the authors to either not present these variables in the method section or present the results associated with them.**

The sexual orientation variable included two modalities: heterosexual and homosexual. The variable did not appear significantly in the results of the lexicometric analysis. Following your recommendations, we have chosen to remove this variable

**7. The lexicometric analyses (as well as the interpretation process) should be more thoroughly described as it is not a well-known kind of analysis.**

You make a valid point that lexicometric analysis is not a well-known kind of analysis, and it may be helpful for the authors to provide a more thorough description of their methods and interpretation process. We could include more detailed explanations of the specific techniques used, such as top-down hierarchical clustering (TDHC), similarity analyses, and correspondence factor analysis (CFA). They could also provide more background on lexicometric analysis and its potential benefits and limitations for analyzing qualitative data. This would help readers better understand the methods used in the study and the validity of the results.

**8. The authors specify they proceeded to a thematic analysis/categorical content analysis. Was it an inductive or deductive one? Were there any double-coding involved? If so, I advise the author to provide an intercoder coefficient.**

The thematic analysis/categorical content analysis in this study was inductive, as the themes emerged from the data itself rather than being pre-determined based on a theoretical framework. Regarding double-coding, two independent coders were involved in the analysis, and any discrepancies in coding were discussed and resolved through consensus. However, we did not provide an intercoder coefficient in the paper, and we agree that this would improve the transparency of the analysis. We will take your suggestion into consideration for future research.

**9. More generally, I advise the authors to use a quality checklist (e.g., COREQ) to ensure rigour in their study.**

The COREQ method was used and the answers to the different checklist items were added in an annexed file. The article meets each of the COREQ criteria. We have modified the method part explaining the use of the method.

Results
**1. The authors specify the number of patients in physicians' practice. Does this concern the current or passed pratice?**

Thanks for your comment. The number of patients in the physicians' practice is their current practice and actual practice at the time of the interview. This was specified in the article.

**2. It is unclear to me whether the two participants who did not meet the clinical criteria for a switch were included in the study or not.**

Thanks for your comment. We have specified that these patients were included even though they did not experienced a switch because their view of treatment switching makes sense in terms of the comprehensive approach of the study.

**3. How were patients classified as "adherent", "hesitant" or "opposed"? What was the authors' criteria?**

The criteria used for these classifications were based on patients' own self-reported behavior and beliefs about medication adherence. Patients who reported taking their medication as prescribed were classified as "adherent", while those who reported occasional or frequent non-adherence were classified as "hesitant" or "opposed", respectively. The criteria used for these classifications were based on patients' own self-reported behavior and beliefs about medication adherence.

**4. What was the mean (+ standard deviation) of the interviews' time?**

For clinicians, the average duration of the interviews is 1 hour and 2 minutes, with a standard deviation of 32 minutes and 22 seconds. For PLWH, the average duration of the interviews was 46 minutes with a standard deviation of 14 minutes We have added this information in the the article.

**5. Offering a translation for the appendices to make them fully accessible to all readers seem relevant to me.**

The interviews and analyses were conducted in French. We have chosen to translate only the dendogram since it was at the heart of the lexicometric analysis. On the other hand, it is more complicated to translate all the figures, in particular to be as faithful as possible to the meaning given by the interviewees

**6.Regarding the lexicometric analysis:
a. The authors mention that classes 4, 5 and 6 are "saturated" with medical discourse. Do they mean it as a kind of "data saturation"?**

Thank you for your comment. We wanted to emphasize that in these classes, only the physicians' speeches appear in a meaningful way. We have added a sentence to the article to clarify this point.

**b. To me this part is not enough developed. Each class should be named, their interpretation should be argued and quotations should be presented to ensure credibility (Lincoln & Guba, 1985)**

We have named and further presented the interpretation of the classes. This appears in the article. We have also added a table in the appendix with the name, interpretation and associated citations for each of the classes

**7. P.5 L.150 : "three thematic categories common to both physicians an patients", do the authors mean that both groups were in agreement on these aspects?**

This means that the analyses revealed that patients and physicians addressed these same themes. We precised this in the article.

**8. Ideally (and if the word limit allows it), when an idea refering to opinions found in both patients and professionals is presented, quotations from both of these groups should be presented.**

We have tried to present quotes for each group, but we are constrained by the word limit

**9. As the results pertaining to the lexicometric analysis are way less developped compared to the thematic analysis I wonder what was the added value of this kind of analysis according to the authors?**

We used the lexicometric analysis to identify central patterns or themes within the participants' discourse. This analysis allowed to highlight the most frequent and significant words used by the participants, which in turn revealed the dominant themes in their narratives. This approach provides an overview of the central themes in the data and allows for a more systematic and objective analysis of the language used by participants.

The thematic analysis was used to develop deeper into the experiences of the participants, exploring the nuances and complexities of their narratives. This allowed for a more comprehensive understanding of their experiences, as well as a more in-depth analysis of the data. Therefore, the lexicometric analysis provided an initial overview and allowed us to identify key patterns, which were then explored and refined through the thematic analysis.

**10. I found the discussion of the article very interesting to read but the authors added some results that were not presented in the results section (e.g., P.10, L.384-386), these information should be first be well presented in this part.**

Thank you for your comment on this point. We have modified the result section and added a section on the comparison between oral dual therapy and injectable treatments.

Minor comments
**1. p.2 l.67 : ARV is written as an acronym while it is the first time the authors mention it. They should write it fully here.**

We have corrected this and clarified the acronym

**2. p.2 l.80 "themes" to describe "topics" broached upon in the interview guide is not the best term as it get mixed up with "themes" resulting from a thematic analysis.**

We have replaced "themes" with "subject areas" for the interview guide to avoid confusion with the themes resulting from a thematic analysis.

**3. Quotations for both the lexicometric and thematic analyses could be presented in a table to respect the word count without compromising on the content of their manuscript.**

We have made a table in the appendix of the different citations for the thematic analysis and the lexicometric analysis. Nevertheless, we wanted to keep some quotations in the body of the text.

**4. The age, gender and status (patients vs. professional) should be specified for each quotation.**

We have added these elements for each quote

**5. "Single-tablet regimens (STR) refer to the quality of life" do the authors mean "refer" or "guarantee" (or something else)?**

We mean that STRs enable better quality of life for patients

**6. There are some typos in the manuscript (e.g., P.6 L. 191 "a subjectively, a better")**

We have corrected these mistakes and the entire document was proofreden.

**7. The title "Results, discussion, conclusions" seems inappropriate.**

We agree that this structure is too schematic. However, the title "Results, discussion, conclusions" is a common structure used in academic writing to organize and present research findings. The aim of this structure is to clearly separate the different sections of the paper and make it easier for all readers to get oriented in the flow of the article.
