# Supplementary material for: Acceptability determinants of a proposal to reduce antiretroviral treatment to an oral two-drug regimen among patients living with HIV and physicians in France
Source: PLoS One. 2024 Sep 30;19(9):e0308784. doi: 10.1371/journal.pone.0308784 (PMC11441690; doi:10.1371/journal.pone.0308784)
Supplement: Attachment — Submitted filename: Response to reviewers.docx [file pone.0308784.s003.docx]

Review Comments to the Author

Please use the space provided to explain your answers to the questions above. You may also include additional comments for the author, including concerns about dual publication, research ethics, or publication ethics. (Please upload your review as an attachment if it exceeds 20,000 characters)

Reviewer #1: (No Response)

Reviewer #3: Dear Authors, it is a privilege to be part of the review process and contribute to the enhancement of such a promising study.
I find the study particularly interesting, especially considering the chosen topic, which has been relatively underexplored thus far. The application of a mixed-method approach adds a valuable dimension to the research, aligning with the evolving trends in the field.

Having reviewed the manuscript, I appreciate the effort that has gone into addressing the comments raised by other reviewers.

I have minor revisions as request in order to further improve the quality of the manuscript.

Minor revisions:

**Title:**

**For clarity, I would suggest adding HIV in the title.**

Thank you for your feedback. The title has been changed as follows: Acceptability determinants of a proposal to reduce antiretroviral treatment to an oral two-drug regimen among patients living with HIV and physicians in France.

**Abstract:**

**Line 9: I would suggest again to indicate “treatment for HIV”.**

We have changed the sentence as follows on line 9 : An oral two-drug regimen (O2DR) in the form of a once-a-day single tablet is now recommended for treatment switching and treatment initiation for HIV.

**Line 12: I would suggest a colon instead of full stop “The study includes 30 interviews: 15….”**

The change was made to line 13 as follows: The study includes 30 interviews : 15 were conducted with doctors caring for people living with HIV, 15 were conducted with patients who had been offered a change of treatment.

**Line 14: I would suggest merging and rephrase the following two sentences to give a clearer information about the double analysis. “A double analysis was carried out on the whole corpus. Lexicometric and thematic analyses made it possible to highlight the structuring of discourses 16 around treatment change and the associated issues in the context of O2DR.”**

We have taken your comment into account. The sentence has been changed on line 14 for greater clarity: A double analysis was carried out: lexicometric analysis to highlight the structuring of the discourse around the change in treatment and a thematic analysis to understand the associated issues more precisely.

**Introduction:**

**Line 64-66: I would specify that the “two oral O2DR have been approved by health authorities” from Europe and North American.**

Nous avons rajouté cette précision à la ligne 59 de la manière suivante : Also, the switch to O2DR has been approved by health authorities from Europe and North American.

**Line 52-59 : Could you specify what PROBI means? I guess it is an acronym**

It is in fact an acronym. For greater clarity, we have defined the acronym PROBI in the introduction on line 12.

**Line 81: To be consistent, I would suggest to not use the word PROBI qualitative “study” since you are presenting the PROBI study as a mixed methods study. Please, keep referring to each quantitative and qualitative part as a component or a phase rather than as a study.**

Thank you for your feedback. For greater consistency, we have taken your suggestion into account by using the term phase rather than study in the corresponding lines (61 and 66) and this has been standardised in the article.

**Methods:**

**It would be interesting to have more information about the kind of health facilities where both physicians and patients were recruited since in other countries the “prise en charge” of HIV patients can be realised in different services/settings.**

Thank you for your comment, these are hospital centers. We have specified the centers from which the participants in lines 75 and 77 were recruited.

**Line 124: I would avoid terms like “the ideal proportion”.**

We have changed the term. The modified sentence is as follows line 89 : Attention was paid to the representativeness of the profiles.

**Line 130: Please add ref for the COREQ checklist.**

We have made a reference in the text to the corresponding appendix line 144.

**Line 153: To make the methodology clearer, I would suggest adding a brief sentence explaining the purpose of the lexicometric analysis.**

Following your feedback, we have proposed an explanatory sentence to introduce the methodology. The modified sentence is on line 118: The lexicometric analysis, thanks to class distribution, made it possible to highlight the structures of the speeches and the prevalence of the themes mentioned by the participants.

**Results:**

**Line 181-2 Please, rephrase the sentence “The number of patients in the physicians' practice is their current practice and active practice at the time of the interview” is not clear.**

Thank you for that clarification. We preferred the term active patient list for greater clarity. The sentence has been changed on line 146: The number of patients corresponds to their current patient list at the time of the interview.

**Line 189: The median time since HIV diagnosis was 21 years [30, 1], the IQR is uncorrect**

Thank you for your feedback, there was indeed an error in the calculation of the median. The calculation has been corrected on line 154

**Line 193-194: the reason of the “exception” is not clear.**

We're sorry, but we can't see what exception is being referred to.
