# Supplementary material for: Acceptability determinants of a proposal to reduce antiretroviral treatment to an oral two-drug regimen among patients living with HIV and physicians in France
Source: PLoS One. 2024 Sep 30;19(9):e0308784. doi: 10.1371/journal.pone.0308784 (PMC11441690; doi:10.1371/journal.pone.0308784)
Supplement: Attachment — Submitted filename: Response to reviewers.docx [file pone.0308784.s004.docx]

Review Comments to the Author

Please use the space provided to explain your answers to the questions above. You may also include additional comments for the author, including concerns about dual publication, research ethics, or publication ethics. (Please upload your review as an attachment if it exceeds 20,000 characters)

Reviewer #3: Dear authors,
I wanted to extend my appreciation for your response to the comments I provided on your manuscript.
After reviewing your revisions, I am pleased to confirm that you have addressed all of the concerns and suggestions I raised.
I commend your efforts and believe that the revisions have significantly strengthened the manuscript.
Best regards

Reviewer #4: I appreciate the opportunity to review this manuscript and would like to reiterate previous reviewers’ comments that the manuscript is interesting, well-written, and methodologically sound. I am somewhat surprised that the authors chose to submit this manuscript to PLOS One rather than a specialized journal focused on HIV and/or qualitative research. Nevertheless, articles in PLOS One undergo rigorous peer review and tend to receive wider exposure, which, from my personal experience, can be beneficial. In my opinion, the authors have carefully and thoroughly addressed previous reviewers’ comments; therefore, I do not believe acceptance should be delayed further. However, I have some minor suggestions that could potentially improve the manuscript. Whether or not the authors decide to implement these changes should not affect the manuscript’s suitability for publication.

Tout simplement, je cherche des poux.

**Dear Reviewer,**

**Thank you for your thoughtful and positive feedback on our manuscript. We appreciate your recognition of the manuscript's quality and your acknowledgment of our efforts in addressing previous reviewers' comments. Regarding your observation about our choice of journal, we chose PLOS One for its broad audience and the rigorous peer review process, which we believe will benefit the dissemination and impact of our research.**

Supplementary information

1. The tables in the supplementary information file are not presented in sequential order. Notably, there are two instances labelled as “Table 3”, which could lead to confusion. I would suggest that the authors carefully review their table captions and make the necessary amendments to their manuscript, ensuring that tables are properly referenced.

**Thank you for your meticulous review and for pointing out the issue with the table captions in the supplementary information file.**

**We have carefully reviewed and corrected the table captions to ensure they are presented in sequential order. The duplicate "Table 3" labels have been addressed, and all tables are now properly referenced. These modifications have been made in the supporting information file attached to the revised manuscript.**

2. The acronyms (e.g., HP, UP-HP) and terms (e.g., adhere, hesitant, opposed) used in the tables are not defined in the caption. Tables should be self-explanatory and comprehensible on their own. I recommend that the authors include definitions for these acronyms and terms within the table captions (or footnotes) to improve readability and understanding without necessitating reference to the main text.

**We have added footnotes to the tables in the supporting information file to define the acronyms (e.g., HP, UP-HP) and terms (e.g., adhere, hesitant, opposed).**

3. I recognize the challenges associated with translating the similarity analysis (bi-therapy and total corpus) from French, particularly in preserving the original meanings conveyed by the interviewees. However, the figure captions currently lack comprehensive descriptions, making them difficult to interpret independently. I suggest that the authors provide more detailed descriptions of the figures, including the principal words/themes identified. This would make the figures more accessible and informative, especially for non-French speaking readers, allowing them to benefit from the insights conveyed.

**We have added further clarifications regarding the figures mentioned in lines 172 to 175. These additions aim to provide a clearer understanding and improve the overall clarity of our manuscript.**

4. The sociodemographic data presented, particularly concerning the patients, is quite limited. I would like to know what type of information was collected through the online self-reported questionnaire prior to the interviews. Comprehensive sociodemographic data are essential for contextualizing the findings, as the acceptability of switching to once-daily regimens (O2DR) may differ significantly based on individual lived experiences. For instance, racial and ethnic disparities exist along the HIV care continuum, and the perspectives of people of color living with HIV may vary from those of other key populations (ex: white MSM, PWID). This could also have programmatic implications. If the authors have chosen not to present this data, it is crucial that this decision be explicitly mentioned in the manuscript as a limitation of the study.

**Thank you for your insightful comments and for highlighting the importance of comprehensive sociodemographic data.We have presented all the sociodemographic data that we collected through the online self-reported questionnaire prior to the interviews. Unfortunately, we did not collect data on participants' ethnic origins, as this information was not included in our initial submission to the ethics committee. We acknowledge that this omission is a limitation of our study.**

Main text

1. The authors have chosen not to include opinions common to both patients and physicians, citing word limit constraints as the reason. However, it’s important to note that PLOS One does not impose a word count limit. I encourage the authors – and the Editor – to use the space needed to fully present themes and ideas, particularly those supported by both patient and physician perspectives. Qualitative and mixed-method studies often require more extensive discussion to thoroughly explore concepts and results. I highly recommend that the authors reconsider their approach if possible

**Thank you for your feedback and for highlighting the importance of presenting themes and ideas supported by both patient and physician perspectives.We analyzed the interviews separately to capture the specific nuances and unique insights from each group. We prefer to present distinct analyses to highlight the particularities of each perspective. This approach allows us to thoroughly explore the specific themes relevant to patients and physicians individually.However, we understand the value of integrating common themes and will consider revisiting our approach in future research.**
